# Extreme rainfall affects assembly of the root‐associated fungal community

**DOI:** 10.1111/nph.14990

**Published:** 2018-01-19

**Authors:** Christopher J. Barnes, Christopher J. van der Gast, Niall P. McNamara, Rebecca Rowe, Gary D. Bending

**Affiliations:** ^1^ School of Life Sciences University of Warwick Gibbet Hill Campus Coventry CV4 7AL UK; ^2^ School of Healthcare Science Manchester Metropolitan University Manchester M1 5GD UK; ^3^ NERC Centre for Ecology & Hydrology Lancaster Environment Centre Library Avenue Bailrigg Lancaster LA1 4AP UK; ^4^ Present address: National History Museum of Denmar University of Copenhagen 83 Sølvgade Madison 1800 Denmark

**Keywords:** extreme weather, mycorrhizal fungi, root‐associated fungi, soil fungi, temporal variation in microbial communities

## Abstract

- Global warming is resulting in increased frequency of weather extremes. Root‐associated fungi play important roles in terrestrial biogeochemical cycling processes, but the way in which they are affected by extreme weather is unclear. Here, we performed long‐term field monitoring of the root‐associated fungus community of a short rotation coppice willow plantation, and compared community dynamics before and after a once in 100 yr rainfall event that occurred in the UK in 2012.
- Monitoring of the root‐associated fungi was performed over a 3‐yr period by metabarcoding the fungal internal transcribed spacer (ITS) region. Repeated soil testing and continuous climatic monitoring supplemented community data, and the relative effects of environmental and temporal variation were determined on the root‐associated fungal community.
- Soil saturation and surface water were recorded throughout the early growing season of 2012, following extreme rainfall. This was associated with a crash in the richness and relative abundance of ectomycorrhizal fungi, with each declining by over 50%. Richness and relative abundance of saprophytes and pathogens increased.
- We conclude that extreme rainfall events may be important yet overlooked determinants of root‐associated fungal community assembly. Given the integral role of ectomycorrhizal fungi in biogeochemical cycles, these events may have considerable impacts upon the functioning of terrestrial ecosystems.

## Abstract 2

See also the Commentary on this article by https://doi.org/10.1111/nph.15086.

## Introduction

There is mounting evidence that global warming is directly increasing the frequency of weather extremes, including heavy rainfall events, droughts and high temperatures across Europe and North America (Mallakpour & Villarini, 2015; Shepherd, 2015), which may be further increased during El Nino events (Cai *et al*., 2014). Terrestrial ecosystems play a key role in determining global climate–ecosystem feedbacks, due to their role in the exchange of greenhouse gases (GHG) including CO_2_, methane and nitrous oxide, and storage of carbon (C) in soil and vegetation. Extreme weather may have major effects on climate–terrestrial ecosystem interactions, with links to reductions of C stocks through a range of feedbacks operating on plant productivity and soil processes (Reichstein *et al*., 2013). However, studies of the impacts of extreme weather events on soil systems are limited due to their rarity and our inability to capture such events in space and time. As a result, very little is known of the ways in which extreme weather, particularly extreme rainfall events, affect soil communities and associated processes, and the consequences for climate–ecosystem feedbacks (Heimann & Reichstein, 2008).

Plants live in close association with distinct microbial communities in the rhizosphere, the radial gradient spanning from the plant root into the soil (Hartmann *et al*., 2008) into which microorganisms are selectively recruited (Berendsen *et al*., 2012). These root‐associated communities are diverse, and although most attention has focused on their bacterial and fungal communities (Smit *et al*., 1999; Smalla *et al*., 2001), they include a plethora of other components including protists and nematodes (Bonkowski, 2004). These communities likely play key roles in terrestrial ecosystems, connecting above‐ and belowground diversity (Bardgett & van der Putten, 2014).

Mycorrhizal fungi, particularly ectomycorrhizal (ECM) and arbuscular mycorrhizal (AM) types are some of the best‐characterized fungal inhabitants of the rhizosphere. These fungi form mutualistic symbioses with their host plants (Smith & Read, 2010), conferring a range of benefits, particularly by increasing host uptake of nutrients (Garcia *et al*., 2014) and providing disease resistance in exchange for C (Chakravarty & Unestam, 1987; Liu *et al*., 2007). These root‐associated fungi can be major components of terrestrial ecosystems through their role in driving biogeochemical cycles (Högberg *et al*., 2010). For example within temperate forest soils, they may contribute a third of ecosystem C and nitrogen (N) mineralization (Finzi *et al*., 2015). ECM dominate the root zone in temperate and boreal forest systems, supporting plant nutrition and productivity while acting as major conduits by which C flows from the plant to the soil on a global scale (Leake *et al*., 2004). Nonmycorrhizal fungi also inhabit the rhizosphere, but their diversity and functional significance are typically less well understood (Kubartová *et al*., 2008; Dean *et al*., 2012). Some such fungi occur as endophytes within the root and can also confer growth benefits to the host plant, whilst saprophytes promote mineralization processes, altering nutrient availability and indirectly influencing plant growth (Smith & Read, 2010; Bardgett & van der Putten, 2014). Pathogens, meanwhile, can directly reduce plant growth and development. Even within each of these differing ‘life strategies’, fungi can also show considerable functional variability (Kiers *et al*., 2011; Dean *et al*., 2012), and changes in the composition of root‐associated fungal communities may have profound ecosystem‐level effects on soil processes such as C, N and phosphorus (P) cycles within natural and agricultural systems.

Assembly of root‐associated fungi has been shown to be regulated by a wide range of spatial and temporal variables. Soil pH has a near ubiquitous effect on soil microbial community assembly (Coughlan *et al*., 2000; Gosling *et al*., 2013; Tedersoo *et al*., 2014), whilst available P and N have been linked to changes in the composition of root‐associated fungal communities, with increases of both linked to reduced mycorrhizal abundance (Gosling *et al*., 2013). Climatic factors such as rainfall and temperature also have been indicated to drive fungal richness on a global level (Swaty *et al*., 1998), whilst increasing geographical distance between soil fungal communities has been linked with increasing dissimilarity, independent to that of changing environmental factors (Lilleskov *et al*., 2004; Barnes *et al*., 2016b). Root‐associated fungal communities also change over time, with strong evidence for seasonality of community structure (Bohrer *et al*., 2004; Dumbrell *et al*., 2011), and inter‐annual shifts which can take place across multiple years (Last *et al*., 1984; Visser, 1995; Daniell *et al*., 2001; Husband *et al*., 2002), adding further complexity to understanding the factors that regulate community assembly.

Given that soil biodiversity and functioning remains largely ‘a black box’, it is unsurprising that to date little is known of the way in which extreme weather affects the assembly of root‐associated fungi, and the consequences for climate–ecosystem feedbacks (Bardgett *et al*., 2008). The data that are currently available on extreme weather–soil interactions are limited and derived from experimental manipulations (Barnard *et al*., 2013; Amend *et al*., 2016); however, drought and rewetting of soil cores has revealed a high resilience of the fungal community to desiccation. Soil saturation following intense rainfall can have profound effects on plant productivity, and exposure for long periods can even cause plant death (Rivest *et al*., 2013). Furthermore, soil saturation leads to hypoxic or anoxic conditions, promoting microbial reduction reactions, and in turn influencing the abundance and composition of soil microbiota (Coutts & Nicoll, 1990; Unger *et al*., 2009; Wilson *et al*., 2011; Wagner *et al*., 2015). Fungi can have varied responses to waterlogging within terrestrial ecosystems, with ‘high moisture’ specialist AMF and saprophytic species that are seemingly unaffected or even thrive in waterlogged and anoxic conditions (Camy *et al*., 2003; Fougnies *et al*., 2007; García *et al*., 2008; Yang *et al*., 2016). ECM fungal species are known to have variable hydrophobicity (Lilleskov *et al*., 2011), which may be an adaptation to moisture amounts within habitats and across seasons (Unestam & Sun, 1995). Some ECM fungi inhabit environments which are permanently waterlogged (Baar *et al*., 2002). However, increasing saturation may reduce ECM fungal abundance across ecosystems (Tedersoo *et al*., 2009), and even induce death of ECM fungal mycelium within an ecosystem as soil becomes waterlogged (Coutts & Nicoll, 1990). However, the way in which change in weather, and in particular weather extremes, affects the composition and function of the rhizosphere fungal community remains unknown, representing a considerable obstacle in understanding future climate‐ecosystem feedbacks.

Here, we quantify the relative effects of spatial (geographical distance between sampling locations), environmental (soil and climatic properties) and temporal variation (seasonal and interannual) in determining the root‐associated fungal community of willow over a 3‐yr period. Soil cores were taken across line transects in October 2010, July 2011, October 2011, July 2012 and October 2012. Fine roots were extracted from soil cores and their associated fungal communities were profiled by high‐throughput sequencing. Significantly, sampling in 2012 coincided with record levels of rainfall across the UK, and locally at our field site, rainfall was at its highest annual levels since records began in 1947. This provided an unique opportunity to elucidate the magnitude to which extreme rainfall and associated soil saturation influences assembly of communities *in situ*, including the responses of fungi with distinct nutritional modes (ectomycorrhiza, pathogens, endophytes and saprophytes), relative to spatial and seasonal variation in soil and climatic factors.

## Materials and Methods

### Study site and experimental design

The study was performed in a short rotation coppice willow plantation near Lincoln, UK (53.3163°N, 0.5777°W), which was subject to minimal land management practices. The site had a 30‐yr mean air temperature of 9.9°C and a fine loam over clay soil (15% clay, 49% sand and 36% silt). In 2000, the willow was planted at a density of 15 000 stools ha^−1^, covering an area of *c*. 9.44 ha. Salix cuttings were planted in paired rows 0.75 m apart, with 1.5 m spaces between the rows. Six closely related *Salix viminalis* L. genotypes were planted in order to limit disease spread, with Tora (60%) being the most abundant (the others being Bjorn (10%), Bowles Hybrid (10%), Jorr (10%) and Jorunn (10%)). Willow genotypic variation was assessed along the line of the trees where line transects were performed, and no pattern was observed in planting (data not shown). Given the density of trees found within the field site, and that roots can spread over 9 m from individual *S. viminalis* trees (Phillips *et al*., 2014), roots were considered an homogenous mixture of multiple genotypes within the upper soil profile. Coppicing began in 2001, with further events in 2004, 2007 and 2010, providing an average yield of 6.72 t ha^−1^. In February 2010, 660 kg ha^−1^ PK fertilizer (Fibrophos, Melton, UK), 20 t ha^−1^ of lime and 20 t ha^−1^ of locally sourced green waste compost were added to the field site; no exogenous nutrient inputs were added during the duration of the experiment. After planting, soil remained untilled throughout the lifetime of the crop. Therefore, soil microbial communities developed over this period without major disruption of the soil matrix, in a system with limited aboveground diversity.

Sampling was performed along single line transects (as per Barnes *et al*., 2016b) at each sampling time. Starting from the southernmost edge of the field heading due north, single line transects started 25 m into the field in order to avoid edge effects, sampling eight locations every 20 m along the line. At each location, four subsamples were taken 1 m apart from north, south, east and west directions from the central location, using a 4.5‐cm diameter soil auger (Van Walt Equipment, Haslemere, UK) to a depth of 15 cm, which was wiped clean with ethanol between samples to limit contamination. Transects were taken in October 2010, July 2011, October 2011, July 2012 and October 2012, shifting a further 3 m north each time in order to avoid sampling of previously disturbed areas.

### Soil nutrient analysis

Soil cores were homogenized evenly by gloved hand, before 100 g was removed and loosely covered to air‐dry, and sieved to < 2 mm particle size. Soil pH and nutrient analysis were performed as described previously in Barnes *et al*. (2016a), with pH, NO_3_, Mg, available K and available P determined for each individual subsample (32 per transect) and averaged at each of the eight sampling locations. This was repeated for each line transect performed at the different times. These soil properties were analysed as they have repeatedly been shown to influence root‐associated fungal community assembly (Geisseler & Scow, 2014).

### Root‐associated fungal DNA extraction

Following removal of *c*. 100 g (from a total of between 200 and 300 g) of soil for nutrient analysis, the remaining soil from each subsample was soaked in deionized water at room temperature for 1 h before all roots were hand extracted using forceps (between 2 and 5 g of roots per subsample). Nonsenescent fine roots (< 2 mm in diameter), which were identified by their colour, texture, turgor and branching structures, were washed over a 6‐mm sieve to remove adherent soil. By selecting these nonsenescent fine roots, sampling of contaminating roots from the herbaceous understory species was also avoided. Entire pools of selected roots were cut into 1‐cm lengths, mixed, and a 0.5 g randomly selected subsample was used for DNA extraction. Roots were initially exposed to mechanical lysis of two periods of 30 s at 30 hz using a TissueLyser (Qiagen) before extraction using the PowerSoil DNA extraction kit (MP Biomedicals, Cambridge, UK) as per the manufacturer's instructions. After extraction, the four subsamples at each sampling location were equilibrated to 25 ng μl^−1^ using a nanodrop ND1000 (Fischer Scientific, Loughborough, UK) and 10 μl of each was pooled to make the DNA template for each of the eight sampling locations for each sampling time, with 40 samples in total used for sequencing (eight sampling locations, five time points).

### Weather data collection

Meteorological data were provided by a weather station located at the field site. These consisted of a cup anemometer, wind vane, air and wet bulb temperatures (Didcot Instruments AWS, Didcot, UK) and a rain gauge (Rimco, Malton, UK). Data were logged as 30‐min averages with the exception of rainfall, which was logged as the 30 min total. Temperature and rainfall data were measured continuously from April 2010 to December 2012. Measurements of volumetric soil moisture content (SWC; m^3^ m^−3^) were only recorded over the main growth period (June–November). SWC was recorded by two CS616 time domain reflectometer (TDR) probes (Cambell Scientific Inc., Logan, UT, USA), which measure the upper 0.3 m of the soil profile. SWC sensors scanned every 10 s and were logged as 30‐min averages. All meteorological data were recorded using CR10 data loggers (Campbell Scientific, UT, USA).

In order to test the soil saturation point, six plastic 66‐mm diameter and 30‐cm depth tubes were hammered into the soil to remove intact cores. Cores were bagged and returned to the lab, before being cut into 0–15 cm and 15–30 cm sections. Volumetric moisture content (VMC) was determined as per Laird *et al*. (2010), with cores saturated within a solution of 0.001 M CaCl_2_ and weighed, before being incubated in an oven until completely dry and reweighed. VMC was subsequently determined as a percentage of bulk density of soil.

### High‐throughput amplicon sequencing and processing of sequencing data

DNA extracts underwent sequencing and subsequent bioinformatics as described previously in Barnes *et al*. (2016b) using ITS1F and ITS4 primers (Gardes & Bruns, 1993; Yang *et al*., 2012), before sequencing on a Roche 454 GS Junior pyrosequencer (454 Life Sciences/Roche Applied Biosystems, Nurley, NJ, USA) at Micropathology Ltd (Coventry, UK). Raw sequences were deposited at the NCBI sequence read archive under the accession numbers SRP056724 for October 2010, and SRP062101 for July 2011, October 2011, July 2012 and October 2012.

Operational taxonomic units (OTUs) were picked *de novo* from sequences using the Uclust algorithm at a 97% similarity level, and chimera checked using the Uchime algorithm in *de novo* mode (Edgar, 2010; Edgar *et al*., 2011). Taxonomic assignments were initially made using the UNITE database (27.08.2013 release from the UNITE project) using Blast (with an *e*‐value > 0.001), before OTUs of >0.1% relative abundance underwent a further Blast search against the continuously updated Unite database and taxonomy reassigned if *e*‐value > 0.001. OTUs with ≤3 reads assigned were also removed to limit the effects of sequencing errors and artefacts (Huse *et al*., 2007).

After processing, 76.6% (92 595) of total reads remained, averaging 2341 reads per sample, across 931 OTUs (mean length = 496.1, standard deviation = 22.5). Chao1 estimates were calculated to estimate the theoretical maximum number of OTUs per sampling location (Chao, 1987), revealing that 63.8% of OTUs were detected after rarefaction and many rare OTUs were undetected. Given the issues McMurdie & Holmes, 2014 raise in performing community analyses with variable library sizes, the rarefied OTU table underwent normalization using the DeSeq2 package as part of Qiime (Anders & Huber, 2010). Negative values were considered zeros before data were finally converted to relative abundances (as percentage of normalized read numbers) as per Fernandez *et al*. (2017). The negative binomial model performed within DeSeq2 can simultaneously account for variation in library sizes and biological variability better than simply subsampling to a uniform sequencing depth alone.

Some members of the Ascomycota, Basidiomycota and Zygomycota can form ECM associations or exist as root endophytes, whereas others act as saprophytes or pathogens. Therefore, a likely life strategy (i.e. nutritional mode) was determined for OTUs in a two‐step process. Initially an automated approach was taken through parsing the OTU table through the FUNGuild database (v.1.0) (Tedersoo *et al*., 2014; Nguyen *et al*., 2016), before a secondary round of assignments were made on a manual basis using previous literature findings (Supporting Information Table S1). These were performed on OTUs with family level or greater specificity of taxonomic assignments and consensus of life strategy within the taxonomic unit. Groups considered were ECM fungi, endophytes, pathogens and saprotrophs, whilst other fungi that were very low in OTU richness such as lichen symbionts and yeasts, or those that were not taxonomically assigned at genus level, were not included within the life strategy analyses.

In order to further understand the ECM fungal response to extreme rainfall, ECM fungal OTUs also were divided by exploration type (Agerer, 2001) and by their mycelial hydrophobicity (Lilleskov *et al*., 2011), with extramatrical exploration type divided into contact‐short, contact‐medium and medium‐long groupings as performed by Fernandez *et al*. (2017).

### Statistical analyses

Repeated measures one‐way ANOVAs were performed on climatic data (soil moisture, precipitation and temperature) to test for significant differences between years, whilst two‐way ANOVAs with sampling time (repeated measure) and distance across transects were performed for assessing significant spatio‐temporal variation in soil properties (Oksanen *et al*., 2007).

Repeated measures one‐way ANOVAs were performed on the OTU data for phylum, family, genus, species, life strategy (known ectomycorrhizal, pathogen, saprophyte and endophyte taxa), hydrophobicity and extramatrical exploration type in order to analyse variation between sampling locations (df = 39) and sampling times (df = 4), and post‐hoc (Tukey's honestly significant difference) tests were performed when significant differences were observed. Due to the high number of analyses performed on individual OTU data, *P*‐values from individual OTU analyses were adjusted using Benjami and Hochberg's false discovery rate procedure in order to limit false‐positives, using the p.adjust function within the stats package of R (Benjamini & Hochberg, 1995). Finally, general linear modelling (GLM) was used to ascertain which temporal (time as weeks after sampling) and environmental parameters (soil pH, P, K, Mg, NO_3_ and SWC) significantly explained variance in the relative abundances of individual OTUs that were found to significantly change between time points using repeated measure ANOVAs (Grafen and Hails, 2002). GLM was performed in Xlstat (Addinsoft, Paris, France) with the aim of disentangling the effects of sampling time from that of SWC.

Hellinger transformations downweight rare species and perform well with high‐throughput sequencing datasets in which there are many zero values, and thus were performed on the community data before Bray–Curtis similarity matrices were generated (Bray & Curtis, 1957). Nonmetric multidimensional scaling analysis (nMDS) was performed from this matrix to visualize trends in community similarity. Variation within the overall community and each life strategy group of the root‐associated fungi separately was quantified via PERMANOVA (using the adonis function within R) against temporal (sampling year and season), environmental properties (pH, NO_3_, Mg, K and P) and geographical distance (between sampling locations) using sampling time as the strata (as October 2010, July 2011, October 2011, July 2012 or October 2012). As the order of explanatory variables affects the outcome of PERMANOVA analyses, variables were placed in order of the largest proportion of variation explained before running the final analysis. The nMDS analyses and PERMANOVA were performed using the vegan package of R (v.2.4‐2; Oksanen *et al*., 2007), whilst nMDS and volcano plots were created using the package ggplots2 (v.2.2.1; Wickham, 2016).

## Results

### Edaphic properties

Biogeochemical values were calculated as the mean of the four subsamples at each sampling location, before undergoing two‐way repeated measures ANOVA that used distance across transect as a factor, and sampling time (as October 2010, July 2011, October 2011, July 2012 and October 2012) as a repeated measure. There were strong significant gradients across the transects for pH (df = 7, *F *=* *14.45, *P *<* *0.001), available P (df = 7, *F *=* *3.51, *P *<* *0.001), available K (df = 7, *F *=* *8.59, *P *<* *0.001) and Mg (df = 7, *F *=* *2.74, *P *=* *0.024), all of which were significantly greater in sampling locations 1–3 relative to sampling locations 5–8 at all time points (data not shown). Between sampling times, pH (df = 4, *F *=* *21.61, *P *<* *0.001) was significantly raised in 2011 and 2012, available P decreased significantly after 2010 (df = 4, *F *=* *16.07, *P *<* *0.001), whilst NO_3_ showed significant seasonal variation (df = 4, *F *=* *13.95, *P *<* *0.001), with substantially higher quantities in soil in July, relative to October transects. Available K also varied over time, peaking in October 2010 and July 2012 time points (df = 4, *F *=* *4.14, *P *=* *0.030) (Fig. S1a–e).

### Climatic data

The average daily temperature at the field site varied throughout the years (df = 10, *F *=* *14.1, *P *<* *0.001), from *c*. –1°C in December 2010 to 17°C in July 2012 (Fig. S2a); however, temperature did not vary between years (df = 2, *F *=* *0.325, *P *=* *0.725) despite 2011 having a warmer spring and autumn compared to the other years. Precipitation did not consistently differ between months (Fig. S2b), yet it differed strongly between years (df = 2, *F *=* *3.86, *P *=* *0.036), with 2012 being substantially wetter in April (140 mm per month) and June (130 mm per month), which was over double that of the previous year's averages. SWC was only monitored between June and November, but reflected rainfall data, with 2012 significantly higher than 2010 and 2011 in all months (df = 2, *F *=* *5.47, *P *=* *0.020), but with no apparent consistent monthly differences between years (df = 4, *F *=* *0.669, *P *=* *0.628; Fig. S2c). The average monthly SWC across the entire monitoring period was 0.313 m^3^ m^−3^; however, for 2012 this figure was 0.406 m^3^ m^−3^ and reached as high as 0.484 m^3^ m^−3^ in both June and July 2012. Saturation tests within the laboratory suggested that complete saturation can occur from 0.510 m^3^ m^−3^, and thus in June and July 2012 soil was 94.9% of minimum saturation (Fig. S3). Furthermore, observations in the field showed a considerable presence of standing surface water throughout this period (Fig. S4).

### Investigating spatial, temporal and environmental factors regulating root‐associated fungal assemblage

**Figure 1:**
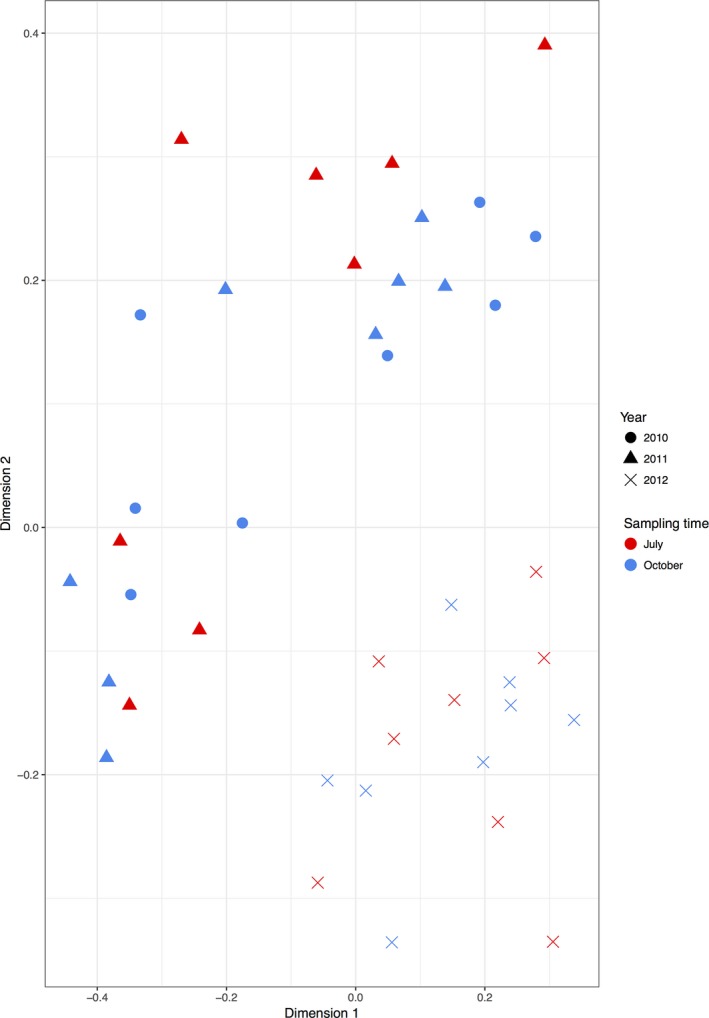
Nonmetric dimensional scaling showing clustering based on similarity of the root‐associated fungal communities between sampling time points.

In order to understand the spatio‐temporal variation of the root‐associated fungal community composition over the sampling period, an initial Bray–Curtis similarity matrix was created from the sequencing data and visualized using multidimensional scaling (limited to two dimensions, stress = 0.145, 999 iterations). Samples taken in 2010 and 2011 grouped together, and were distinct from those taken in 2012 (Fig. 1). There was no evidence for seasonal changes in community composition for either the 2011 or 2012 time points, as there was no grouping of October or July time points within the nMDS ordinations. PERMANOVA was subsequently performed against temporal data (sampling year and season as factors), environmental properties (pH, NO_3_, Mg, K and P) and geographical distance (as location on transect), whilst sampling time point was used as the strata (Table 1). In this analysis, sampling year (2010, 2011 or 2012) had the greatest effect on the community composition, explaining 21.1% of variation, whilst pH explained a further 10.8% of variation (Fig. S5), with a total of 31.9% total community variation explained by measured parameters. There was no significant effect of seasonality (i.e. sampling in either July or October) on community structure over the 3‐yr sampling period. Neither geographical distance between sampling locations nor the remaining soil properties affected community assembly.

**Table 1:** The relative importance of time, geographical distance (between samples) and soil properties for the root‐associated fungal community of willow as revealed by PERMANOVA

Parameter	Degrees of freedom	*F*‐value	*R* ^2^	*P*‐value
Season	1	1.338	0.026	0.155
**Year**	**3**	**3.640**	**0.211**	**0.001**
**pH**	**1**	**5.586**	**0.108**	**0.001**
Distance	1	0.772	0.015	0.736
Nitrate (NO_3_)	1	0.859	0.017	0.612
Magnesium (Mg)	1	0.759	0.015	0.771
Potassium (K)	1	1.045	0.020	0.350
Phosphorus (P)	1	1.356	0.026	0.153
Residuals	29		0.562	
Total	39		1.000	

Bold indicates significance (*P *<* *0.05).

Given the community shift that occurred between 2011 and 2012, community data were partitioned into pre‐ (October 2010, July 2011 and October 2011) and post‐extreme rainfall (July 2012 and October 2012), and reanalysed for seasonal, environmental and edaphic effects that may have been masked by the weather event (Fig. S6; Table S2). The two separate PERMANOVAs revealed that the pre‐extreme rainfall communities were regulated only by pH (21.6%), whilst seasonality (as July or October) and geographical distance (as location on the line transect) had no effect. The post‐extreme rainfall community remained entirely unexplained by all measured parameters.

### Determining the taxonomic shifts associated with the change in community composition in 2012

**Figure 2:**
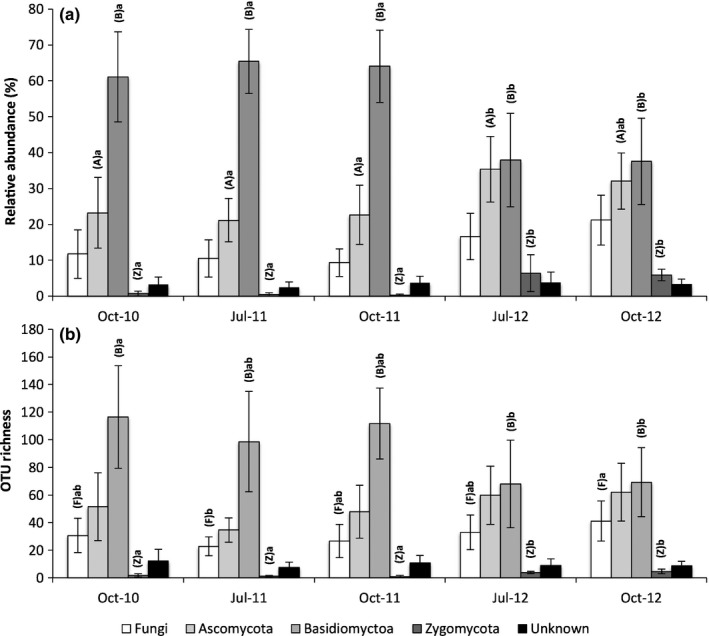
Average (a) relative abundance and (b) operational taxonomic unit (OTU) richness of the willow root‐associated fungal community separated at the phylum level over a 3‐yr sampling period. October 2010, July 2011 and October 2011 were pre‐extreme rainfall at the site, whilst July 2012 and October 2012 were post‐extreme rainfall. Error bars represent ± 1 SD of the mean. Capital letters in parentheses represent the taxonomic groups that differ between sampling times and different lower case letters indicate the associated significant differences, by Tukey's test (α = 0.05).

Total OTU richness varied from 80 to 299 OTUs between samples, and there was no significant difference between sampling times (*F *=* *0.98, *P *=* *0.431). However, the Basidiomycota, which represented the largest phylum by OTU richness, differed significantly between sampling times (*F *=* *4.24, *P *=* *0.008), with between 116.4 and 98.6 OTUs per sampling location recorded across the 2010 and 2011 communities (known as the pre‐extreme rainfall samples hereafter) and only 68.0 and 69.2 in the 2012 communities (known as the post‐extreme rainfall samples hereafter; Fig. 2). The relative abundance of Basidiomycota declined significantly from 65.4% in the pre‐extreme to 37.7% in the post‐extreme rainfall samples (*F *=* *12.27, *P *<* *0.001).

The Ascomycota were the second most OTU‐rich and abundant phylum. Whilst OTU richness ranged from 34.6 to 62.0, there was no significant change over time (*F *=* *2.46, *P *=* *0.068). However, the Ascomycota varied significantly in abundance between time points (*F *=* *5.04, *P *=* *0.004), from 22.3% in the pre‐extreme rainfall samples to 33.7% of the post‐extreme rainfall samples. The Zygomycota were the least abundant and diverse phylum, but OTU richness increased (*F *=* *16.45, *P *<* *0.001) from an average of 1.4 per sampling location in the pre‐extreme to 4.2 per sampling location in the post‐extreme rainfall samples. Furthermore, relative abundance of Zygomycota reads increased significantly (*F *=* *13.06, *P *<* *0.001) from just 0.4% in the pre‐extreme to 6.2% in the post‐extreme rainfall samples.

There were a number of OTU that could only be assigned to the fungal kingdom, or had no taxonomic information at all. They accounted for between 9.3% and 21.2% of reads (*F *=* *5.48, *P *=* *0.002, respectively) and were lowest in July 2012 and highest in October 2012. These averaged 30.8 OTUs per sample and remained stable over time in OTU richness (*F *=* *2.68, *P *=* *0.052). There also was an additional 3.3% average abundance of reads that could not be assigned even at the kingdom level (varying between 2.5% and 3.8% between sampling times), accounting for an average of 9.6 OTUs per sample (ranging from 7.5 and 12.1) which were retained in measures of richness and compositional analyses.

### Determining the functional shifts associated with the change in community composition in 2012

**Figure 3:**
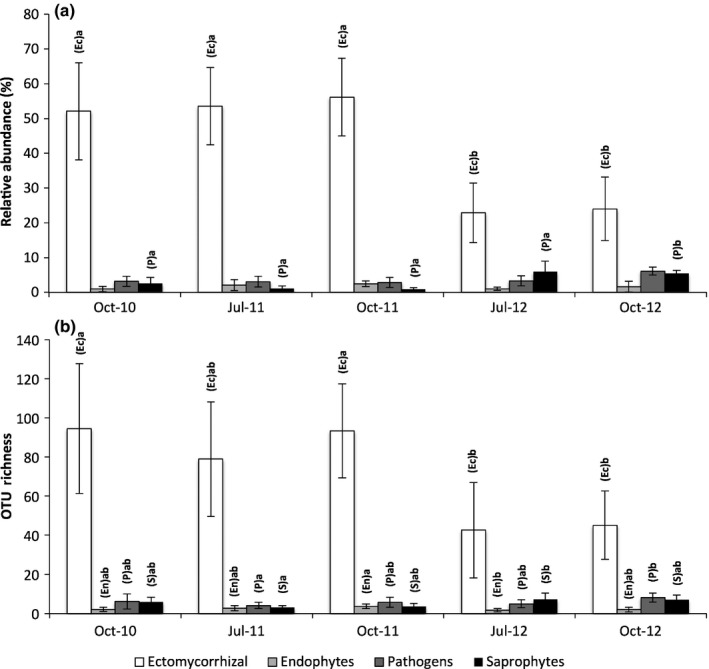
Average (a) relative abundance and (b) operational taxonomic unit (OTU) richness of differing life strategies of root‐associated fungi extracted from willow roots over a 3‐yr sampling period. October 2010, July 2011 and October 2011 were pre‐extreme rainfall at the site, whilst July 2012 and October 2012 were post‐extreme rainfall. Error bars represent ± 1 SD of the mean. Capital letters in parentheses represent the taxonomic groups that differ between sampling times and different lower case letters indicate the associated significant differences, by Tukey's test (α = 0.05).

Relative abundance of ECM fungi remained stable throughout the pre‐extreme rainfall samples, varying from 52.0% to 56.1%. However, there was a significant decline to 23.5% in the samples after the extreme rainfall event (*F *=* *7.71, *P *<* *0.001) (Fig. 3a). There was also a significant decline in ECM fungal OTU richness in the post‐extreme rainfall samples relative to pre‐extreme rainfall samples (*F *=* *20.14, *P *<* *0.001), falling from 79–95 to 43–45 OTUs (Fig. 3b).

Pathogens were the second most abundant and OTU‐rich life strategy, accounting for an average of 3.7% of relative abundance. Pathogen relative abundance differed over time (*F *=* *6.93, *P *<* *0.001), from 3.1% pre‐extreme to 4.7% post‐extreme rainfall. The number of pathogen OTUs within the community varied significantly over time (*F *=* *3.38, *P *=* *0.022), with 5.3 OTUs pre‐extreme and 6.6 OTUs post‐extreme rainfall.

Saprophytes accounted for a further 3.2% of relative abundance and also varied significantly over time (*F *=* *2.94, *P *=* *0.038), ranging from 2.7% and 5.7% in the pre‐extreme and post‐extreme rainfall communities, respectively. Saprophyte OTU richness increased significantly (*F *=* *5.84, *P *=* *0.013) from 5.0% pre‐extreme rainfall to 7.1% afterwards.

Endophytes were consistently found in low abundance and low OTU richness. Endophyte OTU richness varied significantly between sampling points, ranging from an average of 2.5 OTUs pre‐extreme rainfall to 1.9 OTUs post‐extreme rainfall (*F *=* *3.08, *P *=* *0.032), but did not vary in relative abundance, with an average of just 0.56% of total abundance (*F *=* *1.59, *P *=* *0.205).

Closer inspection of ECM fungal families also revealed declines in both OTU richness and abundance in nearly every family. The overall trend of decline in ECM fungal abundance post‐extreme rainfall was driven primarily by changes in the Cortinariaceae (Table S3) whose abundance fell from 32.3% to 9.8% of total relative abundance, whilst OTU richness dropped from 65.5 to 27.5.

**Figure 4:**
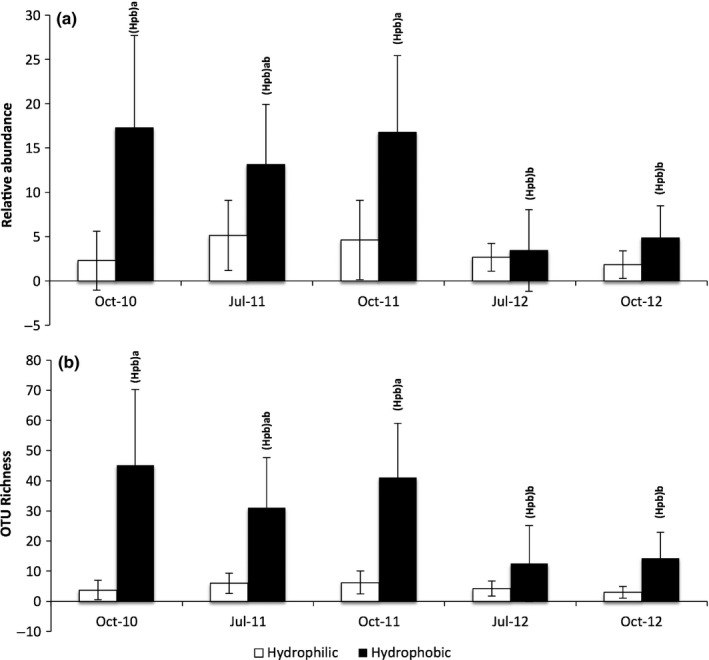
Average (a) relative abundances and (b) operational taxonomic unit (OTU) richness of the ectomycorrhizal (ECM) fungi according to hydrophobicity. ECM fungi were extracted from willow roots over a 3‐yr sampling period. October 2010, July 2011 and October 2011 were pre‐extreme rainfall at the site, whilst July 2012 and October 2012 were post‐extreme rainfall. Error bars represent ± 1 SD of the mean. Capital letters in parentheses represent the taxonomic groups that differ between sampling times and different lower case letters indicate the associated significant differences, by Tukey's test (α = 0.05).

ECM fungi were divided by hydrophobicity and exploration type, and dynamics analysed over time. The hydrophobic ECM fungi were present in considerably greater relative abundance and OTU richness compared to hydrophilic types (Fig 4). Hydrophobic ECM fungi declined significantly in both relative abundance (*F *=* *6.49, *P *<* *0.001) and OTU richness (*F *=* *6.20, *P *<* *0.001) over time, from averages of 15.7% and 39.1 OTUs pre‐extreme rainfall to just 4.1% and 13.4 OTUs, respectively, post‐extreme rainfall. The relative abundance and OTU richness of hydrophilic ECM fungi did not change significantly over time (*F *=* *1.67, *P *=* *0.180 and *F *=* *1.74, *P *=* *0.163, respectively).

There also were significant differences in the contact type of ECM fungi over the sampling period (Fig. S7). The contact‐medium type ECM fungi were the most abundant, and declined significantly in relative abundance (*F *=* *3.26, *P *=* *0.023) but not in OTU richness (*F *=* *6.49, *P *=* *0.131), from averages of 31.6% and 64.1 OTUs pre‐extreme rainfall to 8.4% and 25.5 OTUs, respectively, post‐extreme rainfall. The contact‐short ECM fungi also declined in relative abundance (*F *=* *9.35, *P *<* *0.001), from an average of 5.1% pre‐extreme rainfall to 2.8% post‐extreme rainfall, whilst OTU richness also declined significantly over this period (*F *=* *6.27, *P *<* *0.001), from averages of 6.4 to 4.6 OTU. The long‐range ECM fungi were present in too low abundance and OTU richness to be meaningfully analysed, with an average richness of just 0.1 OTU and relative abundance of 0.1%.

### Investigating the key fungal OTUs within the pre‐ and post‐extreme rainfall root‐associated communities

We further analysed shifts after the extreme rainfall event by performing repeated measure ANOVAs for individual OTUs over the sampling period and visualized this variation using volcano plots (Fig. S8; Newbold *et al*., 2017). In total, 42 OTUs were shown to vary significantly between time points (*q *<* *0.001), with 41 increasing in abundance and only one decreasing in the post‐extreme rainfall samples (Table S4). Despite the overall significant decline in ECM fungal relative abundance and OTU richness, no single ECM fungal OTU declined significantly post‐extreme rainfall at the *q *<* *0.001 level, although nearly all showed a numerical decline in 2012. Meanwhile four pathogens (two *Truncatella angustata* OTUs, a *Plectosphaerella* OTU and a *Pilidium* OTU) and five saprophytic OTU (four *Mortierellaceae* OTUs and a *Sporormiaceae* OTU) increased significantly in abundance. A further four soil yeasts (All *Cryptococcus* OTUs) and two lichenized fungi (*Venturiaceae* OTU and *Verrucaria andesiatica* OTU) also increased in relative abundance, in addition to 26 OTUs with an unknown life strategy. The only OTU to significantly decrease in relative abundance within the post‐extreme rainfall samples could not be assigned to a life strategy.

Finally, GLM was performed on the 42 OTUs that varied significantly between sampling times, with individual OTU abundances analysed against soil pH, P, K, Mg, NO_3_, SWC and time (weeks) (Table S4). As expected, time correlated with 23 of these 42 OTUs, and accounted for the largest percentage of variation of these OTUs, 20.3% on average. However, SWC correlated with more of these 42 OTUs than time, with a total of 33 OTUs correlating with SWC and an average of 16.7% of variation. Whilst every OTU correlated with either time or SWC, or both, soil pH, P, K and Mg correlated with relatively few OTU abundances, with just two, six, five, four and nine OTUs, respectively.

## Discussion

This work demonstrates that a root‐associated fungal community structure was relatively consistent between October 2010 and October 2011, before a dramatic taxonomic and functional transition took place between October 2011 and July 2012, which coincided with an extreme rainfall event that left soils saturated and likely under anoxic soil conditions. As part of this transition in the root‐associated fungal communities, there was a substantial decline in the relative abundance and richness of the ectomycorrhizal (ECM) fungal community, and a concomitant increase in the relative abundance and richness of pathogens and saprophytes. These changes persisted until at least October 2012.

The driver for the community transition between 2011 and 2012 was not directly elucidated. A strong gradient in soil properties existed across transects, which affected composition of the root‐associated fungi (pH) and may have overshadowed any seasonal changes in composition, which have previously been observed in studies of root‐associated fungi (Bohrer *et al*., 2004; Dumbrell *et al*., 2011), and which were not detected here. Previous studies have demonstrated extensively that soil nutrients heavily regulate the composition of root‐associated fungal communities (Klamer *et al*., 2002; van der Gast *et al*., 2011). The field site underwent coppicing in the spring of 2010, alongside fertilization, compost application and liming, which was the most likely cause of the reduction in phosphorus (P) availability between 2010 and the later time points. However, major soil physico‐chemical properties did not differ significantly in 2012 compared to 2010 and 2011, and although a delayed response to the intense management practices of spring 2010, 2 yr earlier, cannot be ruled out, there were no farm management practices imposed during the sampling period, such as crop harvest, fungicide application or tillage that could have directly or indirectly altered root‐associated fungal biota in such a sudden manner.

However, importantly, climate records show that although temperature was comparable between 2010, 2011 and 2012, there were major differences in rainfall between the 2010/2011 and 2012 growing seasons. Heavy rainfall in early 2012 resulted in peak soil moisture content (SWC) field measurements throughout June and July, and a considerable amount of surface water was present at the field site throughout this period (Fig. S4). Nationally, 2012 was the wettest summer, and second wettest year in the UK since records began in 1910 (Parry *et al*., 2013), whilst locally it was the highest rainfall year since records began in 1947 (Waddington monitoring station, MetOffice, UK; *c*. 14 km away from study site). When general linear modelling (GLM) were used to partition variation of the temporally variable operational taxonomic units (OTUs), SWC was the best predictor of OTU abundance, correlating with more OTU than time and all other environmental variables. The taxonomic and functional transition in the root fungal community in 2012 was therefore associated with an extreme weather event, and the extreme rainfall clearly resulted in the prolonged soil saturation we observed during the early summer of 2012.

Soil saturation can result in hypoxic or anoxic soil conditions which can result in changes in soil microbial communities and death of biomass, dependent on the extent and longevity of O_2_ depletion (Wagner *et al*., 2015). Relative to the pre‐extreme rainfall samples, in the post‐extreme rainfall samples ECM fungal OTU richness halved and relative abundance declined by nearly two‐thirds, driven primarily by basidiomycete ECM fungi, and the Cortinariaceae in particular. Laboratory studies have shown that ECM fungus hyphal growth and ECM formation are reduced at high soil moisture contents (Theodorou, 1978; Lodge, 1989; Boucher & Malajczuk, 1990) and within experimental plots, extra‐matrical hyphae of ECM fungi have been shown to die under waterlogged conditions (Coutts & Nicoll, 1990). The mycelia produced by fungal species, particularly ECM fungi, range from hydrophobic to hydrophilic. This may be an adaptation to widely contrasting soil moisture availability between habitats and across seasons, with hydrophobicity an adaptation to retain water in dry soil and prevent inundation in wet soil. Fungal species with hydrophilic mycelium may be better able to resist, or show preference for, water saturated soil (Unestam & Sun, 1995). Importantly, we found that the extreme rainfall in 2012 was associated with losses in richness and relative abundance of hydrophobic but not hydrophilic ECM fungi, with losses from both the contact‐short and contact‐medium exploration types, providing further support that the transitions in community structure which we detected could be the result of soil water saturation in 2012.

Reduced ECM fungal abundance under flooding could have negative implications for plant vitality for a number of reasons: (1) Flooding has been linked to increased nutrient availability to plants (Mendoza *et al*., 2005), and under these conditions, the ECM fungal continuum suggests that ECM fungi could become slightly parasitic to their hosts (Karst *et al*., 2008). (2) ECM fungi show functional diversity, particularly in soil resource uptake and contributions to host nutrition, and thus major community transitions effecting a wide phylogenetic range of ECM fungi, such as that observed here, could impact upon host nutrient uptake (Leake *et al*., 2004). (3) Increased diversity and relative abundance of root pathogens were associated with the rainfall event. Mycorrhizal fungi may play a vital role in protecting plants from attack by pathogens, and increased colonization by pathogens could be a consequence of reductions in the diversity and composition of ECM fungi (Nagy & Fossdal, 2013).

Changes in the abundance and composition of ECM fungi of the magnitude observed in the post‐extreme rainfall samples could represent shifts in the pathway by which carbon (C) passes from the plant to the soil, given that ECM fungi are large sinks of C within forest ecosystems (Durall *et al*., 1994; Högberg *et al*., 2010). Additionally, the increased diversity and relative abundance of saprophytes could also indicate the increased importance of assimilate reaching soil decomposer pathways following the rainfall event, through increased decomposition of root hairs, roots or other root‐associated fungi in response to flooding (Sauter, 2013; Jaiphong *et al*., 2016).

It should be noted that the shift in the ECM fungal community persisted for the duration of the growing season of 2012. Clearly the timescales and extent of community recovery will be of fundamental importance in determining the ecosystem‐level implications and legacy of the extreme weather. This study was conducted within an homogenous willow plantation. There is evidence that ECM fungal richness increases with plant richness (Johnson *et al*., 2005; Tedersoo *et al*., 2014), and the extent to which the resistance and resilience responses of willow‐associated fungal communities reflects that of more diverse natural ecosystems is unclear.

Extensive research into the biotic and abiotic factors regulating root‐associated fungi has been performed (Tedersoo *et al*., 2014), yet the weather‐induced shift in taxonomic and functional composition has, to the best of the authors' knowledge, not been observed before within environmental samples. Studies of the effects of disturbances on soil fungal community composition generally have been limited to persistent changes, such as fertilization (Brearley *et al*., 2007; Gosling *et al*., 2013) and pollution (van der Gast *et al*., 2011; Thion *et al*., 2012). However, precipitation has been linked to changes in AM fungal community composition (Hazard *et al*., 2013) and sporulation (Lovelock *et al*., 2003), and ECM fungal richness (Tedersoo *et al*., 2012), in the field. Studies of the effects of manipulation of soil moisture content on fungal communities are limited to just a handful of studies. Whilst soil fungal communities had considerable resilience to droughts (Evans & Wallenstein, 2012; Barnard *et al*., 2013; Amend *et al*., 2016), increasing soil water content has had mixed results, reducing fungal diversity in a study by Hawkes *et al*. (2011) but having a nominal effect in a study by Fry *et al*. (2016). These results together with those presented in our study indicate that ECM fungal communities are sensitive to precipitation, with pronounced effects at very high soil water contents.

Extreme weather may therefore be an often overlooked regulator in microbial community development over annual time frames, potentially overshadowing the more frequently observed seasonal community variations (Bell *et al*., 2009; Dumbrell *et al*., 2011; Gilbert *et al*., 2012). With projections suggesting increases in the frequency of extreme weather patterns associated with climate change (Hansen *et al*., 2012), and extreme weather already known to impact C cycling, leading to losses in terrestrial C stocks (Reichstein *et al*., 2013), there is a pressing need to understand the impacts of extreme weather events on soil fungal processes and their environmental significance. The decreasing costs of performing large‐scale metagenomic and metatranscriptomic studies should allow the comprehensive long‐term environmental monitoring studies urgently required to understand climate‐rhizosphere feedbacks to be performed.

## Author contributions

G.D.B., C.J.v.d.G. and N.P.M. conceived the study; G.B., C.J.B., N.P.M. and C.J.v.d.G. designed the sampling strategy; C.J.B. performed the sampling and analysis; C.J.v.d.G. advised on spatial statistical methods; R.R. and N.P.M. performed soil saturation tests and provided assistance in the field; C.J.B and G.D.B. wrote the manuscript; and all authors contributed to revisions.

## Supporting information

Please note: Wiley Blackwell are not responsible for the content or functionality of any Supporting Information supplied by the authors. Any queries (other than missing material) should be directed to the *New Phytologist* Central Office.

**Fig. S1 **Boxplots of nutrient content for each sampling time points pH, (b) P, K, Mg and NO_3 _kg^−1^.
**Fig. S2 **Monthly average temperature, monthly precipitation and monthly soil water content from the willow site over the 3‐yr sampling period.
**Fig. S3** Boxplot showing distribution of soil VMC which corresponded with full saturation.
**Fig. S4 **Evidence of surface water found within July 2012 that was not present in any of the other sampling events.
**Fig. S5** Nonmetric dimensional scaling showing clustering based on similarity of the root‐associated fungal communities and pH shown by colour.
**Fig. S6 **Nonmetric dimensional scaling plots with clustering based on similarity of the root‐associated fungal communities, partitioned into before and after extreme rainfall.
**Fig. S7 **Average relative abundances and OTU richness of the ECM fungi divided by exploration type, sampled from willow roots over the 3‐yr period.
**Fig. S8 **Volcano plots of change in relative abundance of each OTU between seasons plotted against ‐log_10_(*q*) values generated from repeated measure ANOVAs (using data from all sampling times).
**Table S2** Relative importance of season, geographical distance (between samples) and soil properties on the root‐associated fungal communities of willow as revealed by PERMANOVA, with the pre‐shift communities (October 2010, July 2011 and October 2012) and post‐shift communities (July 2012 and October 2012) also analysed again in separate analyses
**Table S3 **Average relative abundance and OTU richness of ECM families within SRC willow root‐associated fungal communities sampled over the 3‐yr period

**Table S1** List of taxonomic assignments and references, in addition to *P*‐values (and subsequent *q*‐values) produced via repeated measure ANOVAs for each individual OTU

**Table S4** Table of OTUs which significantly varied in relative abundance over time (as found with repeated measure ANOVAs), whilst GLM models were also performed for each of these OTU to correlate their abundance against time and environmental properties
