# Supplementary material for: Extreme rainfall affects assembly of the root‐associated fungal community
Source: New Phytol. 2018 Jan 19;220(4):1172–84. doi: 10.1111/nph.14990 (PMC6282977; doi:10.1111/nph.14990)
Supplement: Supplementary file 1 — Fig. S1 Boxplots of nutrient content for each sampling time points pH, (b) P, K, Mg and NO3 kg−1. Fig. S2 Monthly average temperature, monthly precipitation and monthly soil water content from the willow site over the 3‐yr sampling period. Fig. S3 Boxplot showing distribution of soil VMC which corresponded with full saturation. Fig. S4 Evidence of surface water found within July 2012 that was not present in any of the other sampling events. Fig. S5 Nonmetric dimensional scaling showing clustering based on similarity of the root‐associated fungal communities and pH shown by colour. Fig. S6 Nonmetric dimensional scaling plots with clustering based on similarity of the root‐associated fungal communities, partitioned into before and after extreme rainfall. Fig. S7 Average relative abundances and OTU richness of the ECM fungi divided by exploration type, sampled from willow roots over the 3‐yr period. Fig. S8 Volcano plots of change in relative abundance of each OTU between seasons plotted against ‐log10(q) values generated from repeated measure ANOVAs (using data from all sampling times). Table S2 Relative importance of season, geographical distance (between samples) and soil properties on the root‐associated fungal communities of willow as revealed by PERMANOVA, with the pre‐shift communities (October 2010, July 2011 and October 2012) and post‐shift communities (July 2012 and October 2012) also analysed again in separate analyses Table S3 Average relative abundance and OTU richness of ECM families within SRC willow root‐associated fungal communities sampled over the 3‐yr period [file NPH-220-1172-s001.pdf]

***New Phytologist* Supporting Information**

Article title: Extreme rainfall affects assembly of the root associated fungal community

Authors: Christopher J. Barnes, Christopher J. van der Gast, Niall P. McNamara, Rebecca Rowe and Gary D. Bending

Article acceptance date: 03 December 2017

The following Supporting Information is available for this article:

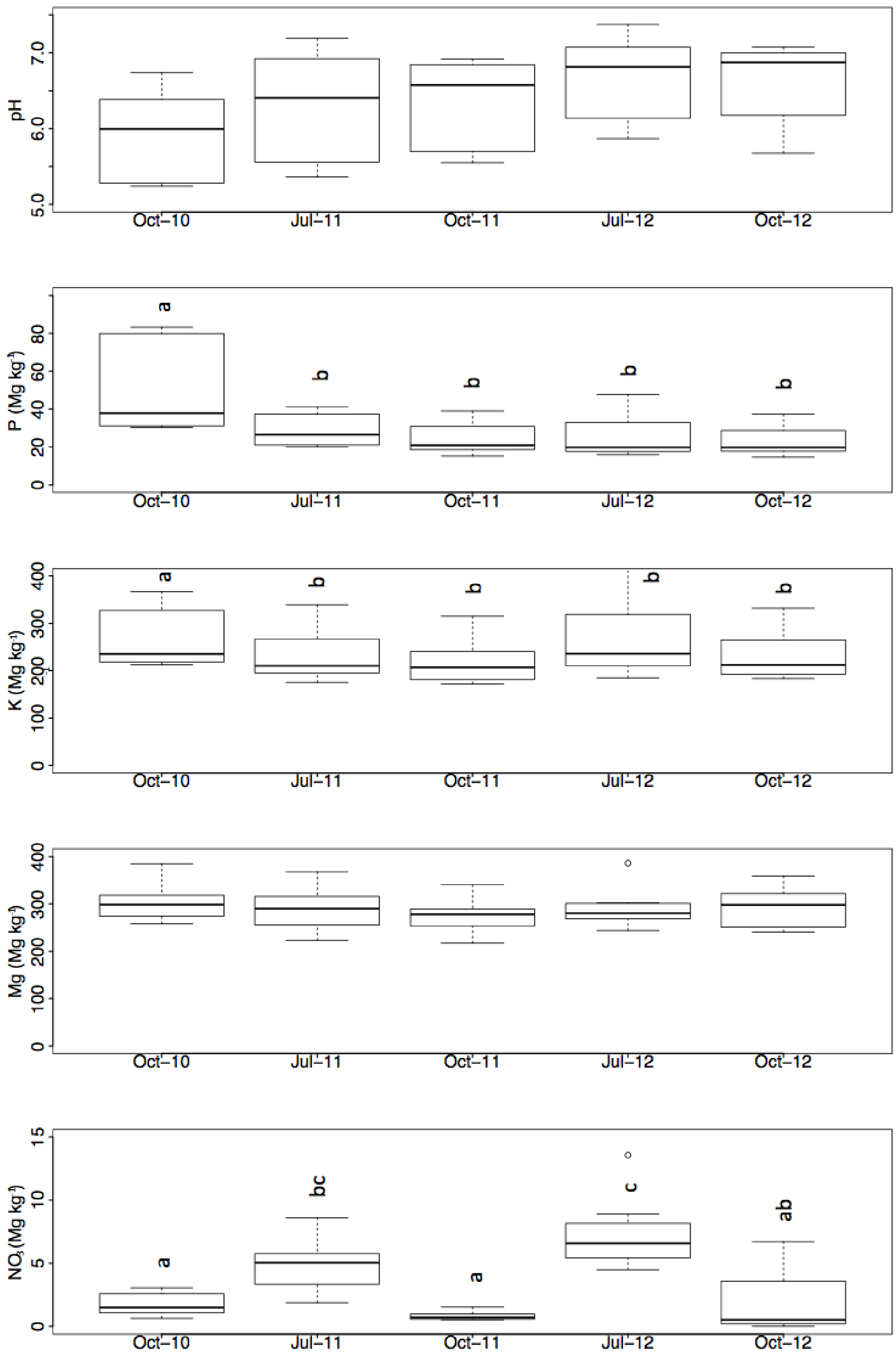


**Fig. S1** Boxplots of nutrient content for each sampling time points (a) pH, (b) P (mg kg$^{-1}$), (c) K (mg kg$^{-1}$), (d) Mg (mg kg$^{-1}$) and (e) $NO_3$ kg$^{-1}$ (mg kg$^{-1}$). Different lower-case letters indicate differences within temporal replicates using Tukey's HSD at α = 0.05.

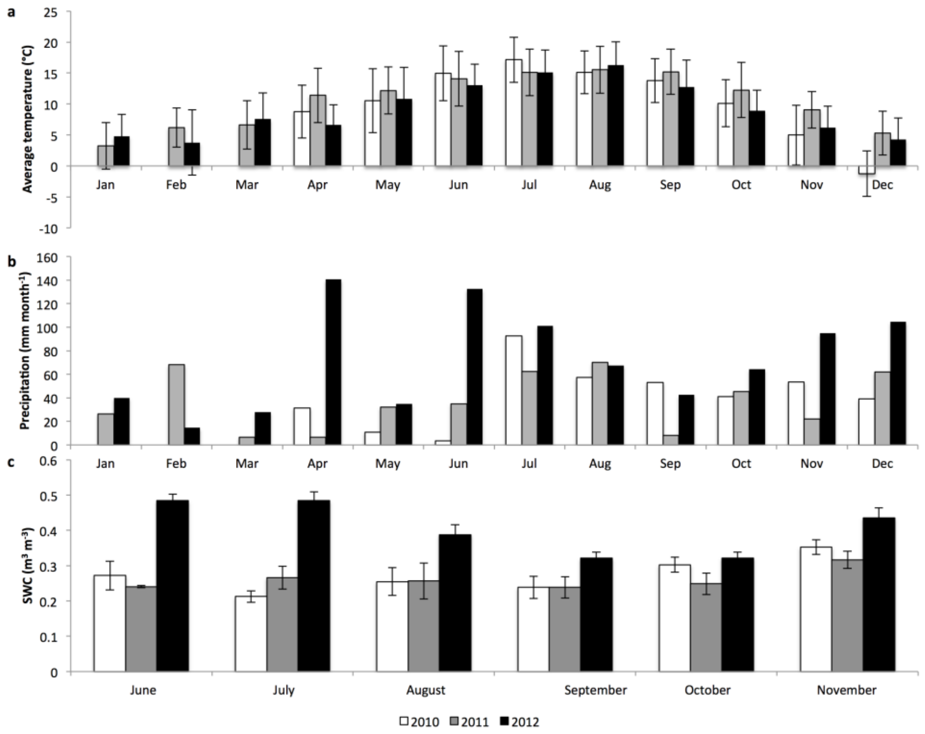


**Fig. S2** Weather data from the willow site over the 3-year sampling period. a.) Monthly average temperature (˚C) b.) Monthly average rainfall (mm of precipitation month$^{-1}$) c.) soil water content (m$^3$ m$^{-3}$) that was only monitored between the June to November period of each year. Error bars represent ±1 standard deviation of the mean.

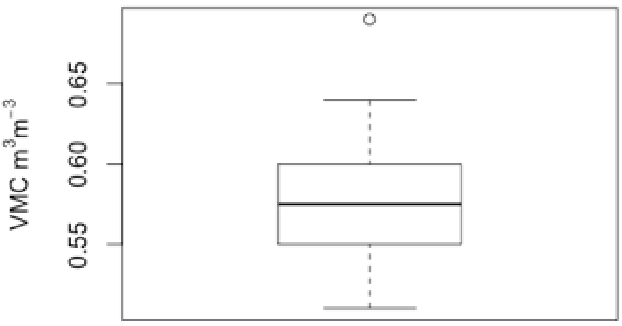


**Fig. S3** Boxplot showing distribution of soil VMC which corresponded with full saturation.

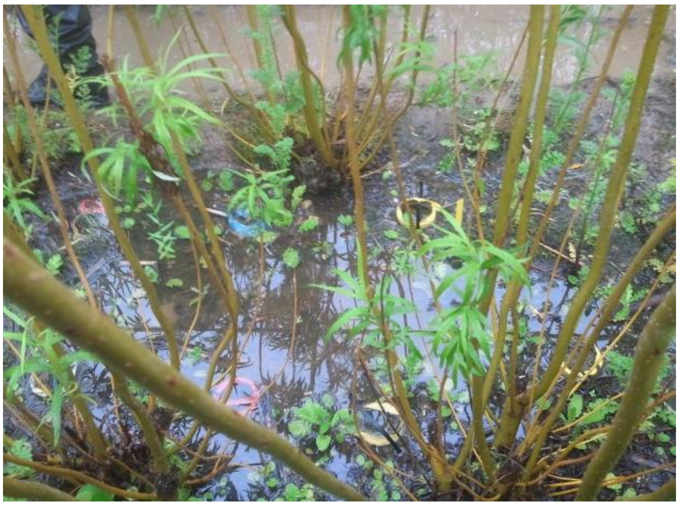

**Fig. S4** Evidence of surface water found within July 2012 that was not present in any other the other sampling events.

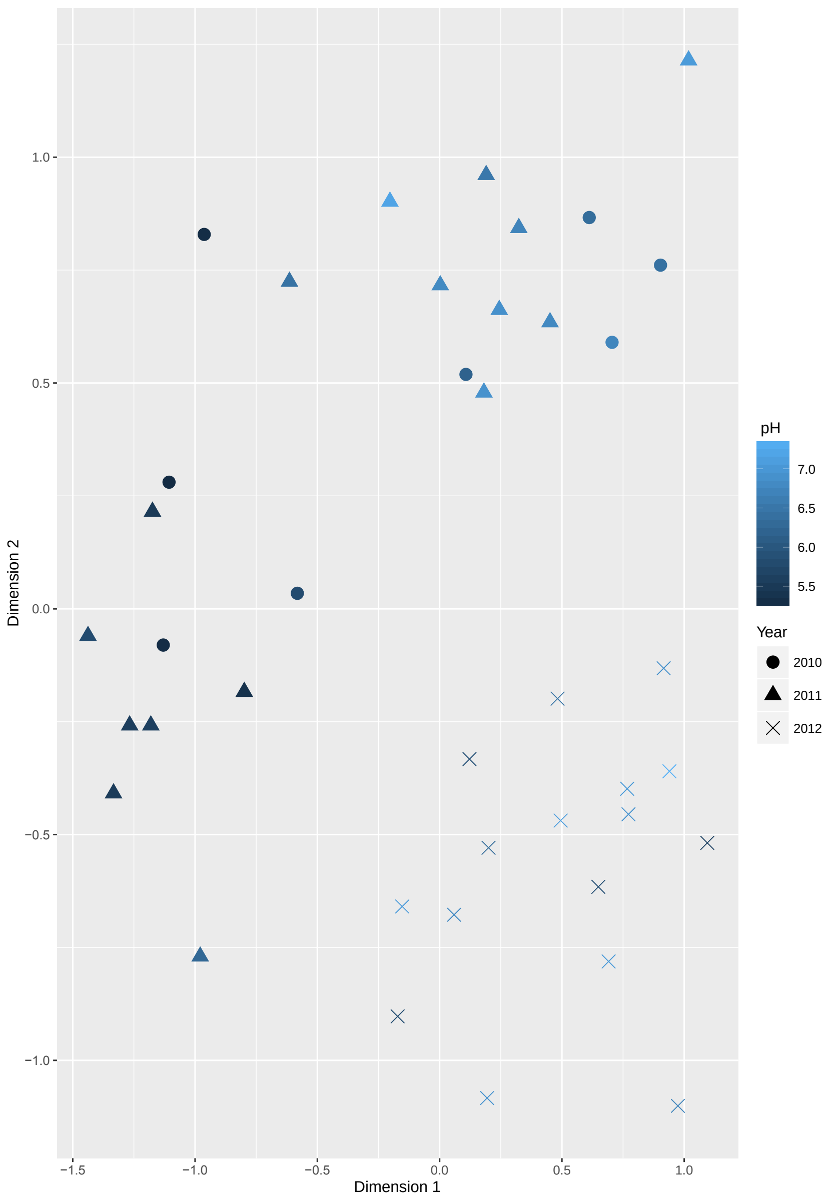


**Fig. S5** Non-metric dimensional scaling showing clustering based on similarity of the root-associated fungal communities and pH shown by colour.

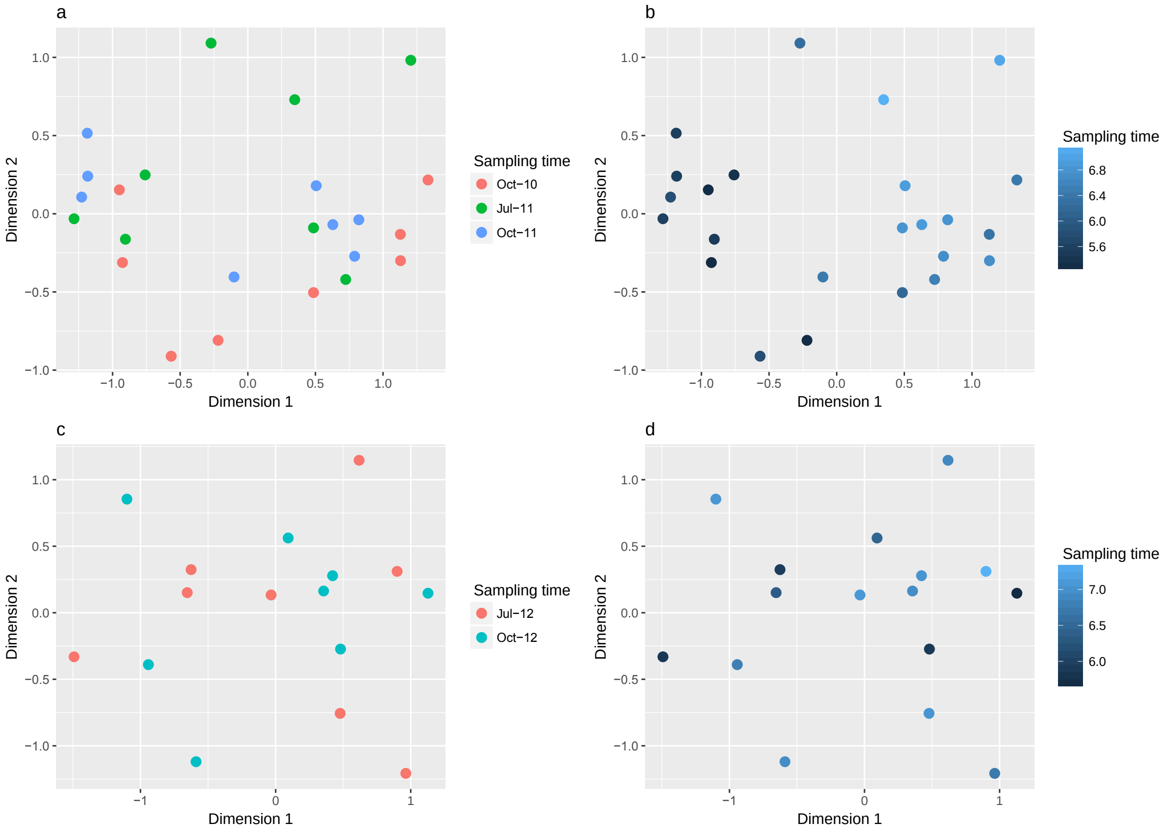


**Fig. S6** Non-metric dimensional scaling showing clustering based on similarity of the root-associated fungal communities. (a) and (b) are the fungal communities of 2010 and, with colour representing sampling time (a) and pH (b). (c) and (d) are the fungal communities of 2012, with colour representing sampling time (a) and pH (b).

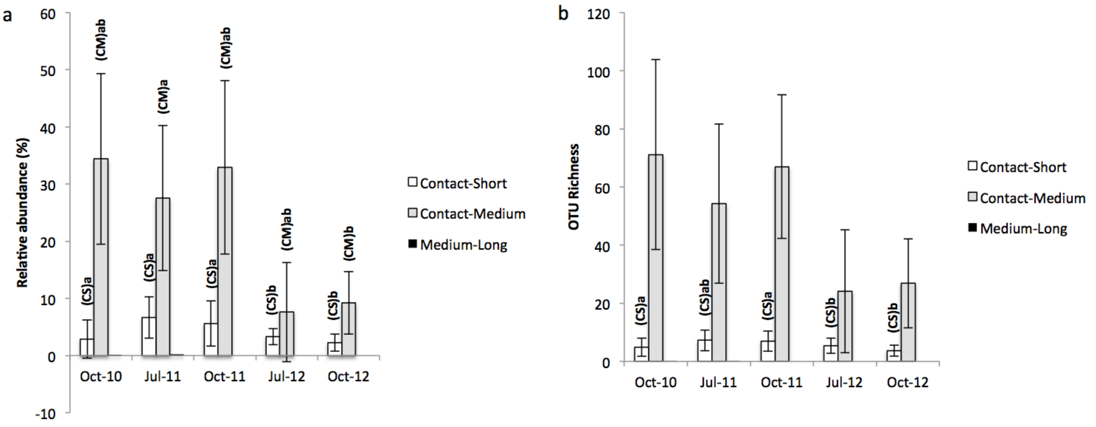


**Fig. S7** Average (a) relative abundances and (b) OTU richness of the ECM fungi divided by exploration type. ECM were extracted from willow roots over the three year sampling period. Error bars represent ±1 standard deviation of the mean. Different letters in parentheses represent the exploration type and lower-case letters indicate differences within temporal replicates using Tukey's HSD at α = 0.05.

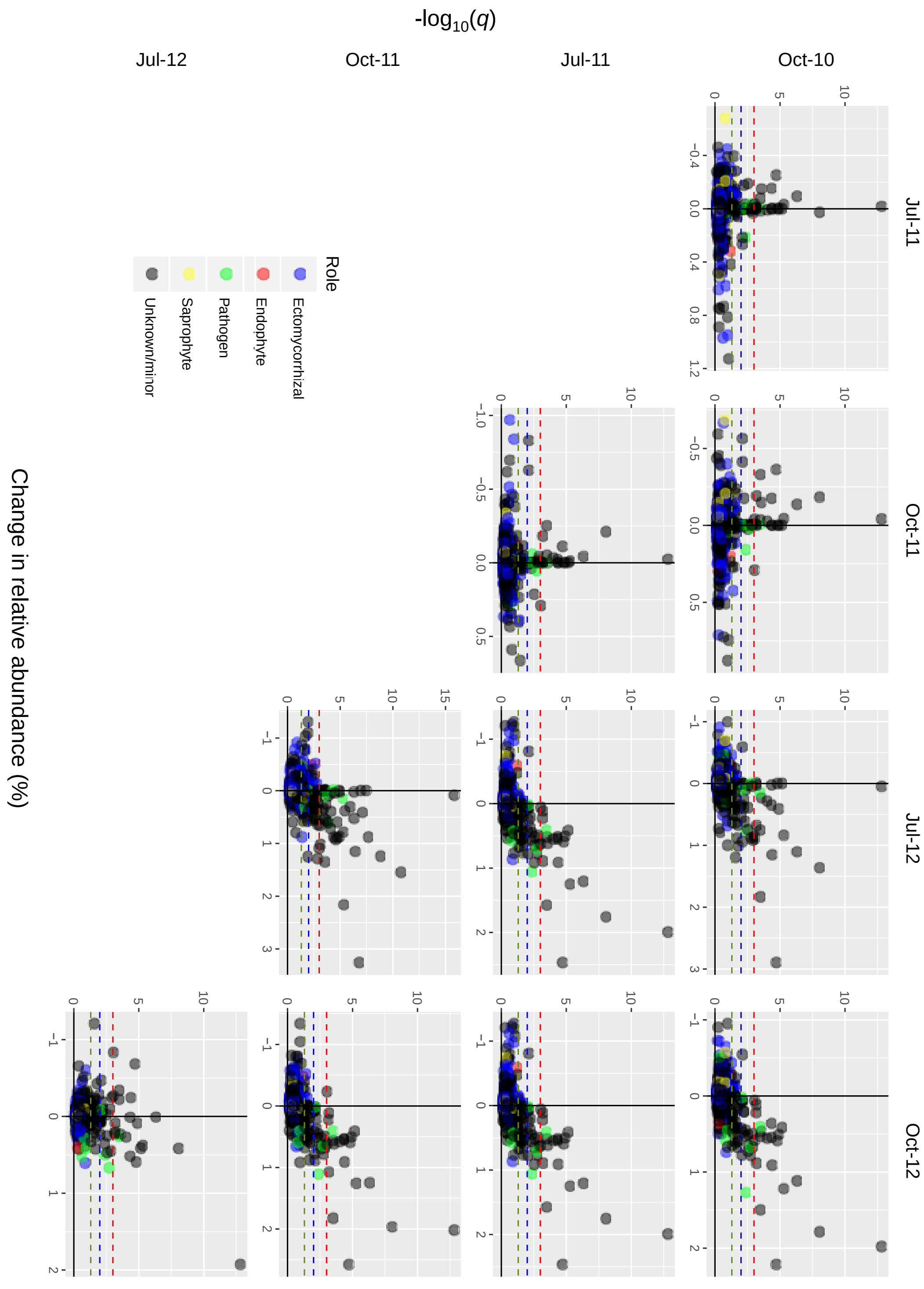


**Fig. S8** Volcano plots of change in relative abundance of each OTU between seasons plotted against -log10($q$) values generated from repeated measure ANOVAs (using data from all sampling times) (Newbold *et al.*, 2017). Negative changes in abundance were considered positive during transformations, before reversion back to negative after the transformation. The dashed red, blue and dark green lines indicates significance at $q$>0.001, $q$>0.01 and $q$>0.05 respectively. For clarity, minor life strategies including soil yeasts and lichenized fungi were included within the unknown fungal group.

**Table S2** Relative importance of season, geographical distance (between samples) and soil properties on the root-associated fungal communities of willow as revealed by PERMANOVA. Given the large shift in composition between the 2011 and 2012 communities, the pre-shift communities (October 2010, July 2011 and October 2012) and post-shift communities (July 2012 and October 2012) were analysed in separate analyses.

**2010 and 2011 samples**

| Parameter | Degrees of freedom | *F*-value | $R^2$ | *P*-value |
|---|---|---|---|---|
| **pH** | 1 | 6.260 | 0.216 | 0.001 |
| Season | 1 | 1.342 | 0.046 | 0.174 |
| Distance | 1 | 1.283 | 0.044 | 0.203 |
| $NO_3$ | 1 | 0.992 | 0.034 | 0.391 |
| Mg | 1 | 0.994 | 0.034 | 0.375 |
| K | 1 | 1.076 | 0.037 | 0.304 |
| P | 1 | 1.064 | 0.037 | 0.313 |
| Residuals | 16 | 0.552 | | |
| Total | 23 | | | |

**2012 samples**

| Parameter | Degrees of freedom | *F*-value | $R^2$ | *P*-value |
|---|---|---|---|---|
| pH | 1 | 0.862 | 0.060 | 0.674 |
| Season | 1 | 1.300 | 0.090 | 0.116 |
| Distance | 1 | 0.867 | 0.060 | 0.688 |
| $NO_3$ | 1 | 0.933 | 0.065 | 0.547 |
| Mg | 1 | 0.782 | 0.054 | 0.820 |
| K | 1 | 0.791 | 0.055 | 0.800 |
| P | 1 | 0.910 | 0.063 | 0.574 |
| Residuals | 8 | 0.554 | | |
| Total | 15 | | | |

**Table S3** Average relative abundance and OTU richness of ECM families within SRC willow root-associated fungal communities sampled over the three year period.

| Family | Family | Oct-10 | | Jul-11 | | Oct-11 | | Jul-12 | | Oct-12 | |
|---|---|---|---|---|---|---|---|---|---|---|---|
| | | Rel. Ab. (%) | OTU Rich. | Rel. Ab. (%) | OTU Rich. | Rel. Ab. (%) | OTU Rich. | Rel. Ab. (%) | OTU Rich. | Rel. Ab. (%) | OTU Rich. |
| Ascomycota | Helvellaceae | 0.28 | 0.50 | 0.23 | 0.38 | 0.18 | 0.50 | 0.25 | 0.50 | 0.21 | 0.75 |
| | Pyronemataceae | 0.15 | 0.38 | 1.14 | 0.75 | 0.35 | 0.63 | 0.01 | 0.13 | 0.11 | 0.38 |
| | Tuberaceae | 1.21 | 3.13 | 2.12 | 2.25 | 1.45 | 2.50 | 0.06 | 0.50 | 0.10 | 0.50 |
| Basidiomycota | Bolbitiaceae | 1.34 | 3.50 | 2.58 | 3.50 | 3.12 | 4.75 | 0.86 | 1.88 | 0.59 | 1.75 |
| | Cortinariaceae | 34.82 | 72.50 | 28.49 | 55.38 | 33.78 | 68.75 | 9.27 | 26.50 | 10.25 | 28.50 |
| | Hymenogasteraceae | 0.00 | 0.00 | 0.08 | 0.25 | 0.13 | 0.13 | 0.00 | 0.00 | 0.02 | 0.25 |
| | Paxillaceae | 0.16 | 0.13 | 0.24 | 0.13 | 0.00 | 0.00 | 0.00 | 0.00 | 0.00 | 0.00 |
| | Russulaceae | 1.80 | 2.00 | 4.15 | 4.75 | 3.59 | 3.88 | 1.02 | 1.88 | 0.82 | 1.38 |
| | Sebacinales | 4.37 | 4.13 | 5.13 | 3.50 | 3.85 | 3.13 | 3.80 | 3.00 | 3.88 | 3.25 |
| | Thelephoraceae | 0.08 | 0.38 | 0.02 | 0.13 | 0.14 | 0.63 | 0.00 | 0.00 | 0.00 | 0.00 |

## References

**Newbold LK, Burthe SJ, Oliver AE, Gweon HS, Barnes CJ, Daunt F, van der Gast. (2017).** Helminth burden and ecological factors associated with alterations in wild host gastrointestinal microbiota. *ISME J* **11**: 663–675.
